# Photodynamic Therapy: A Prospective Therapeutic Approach for Viral Infections and Induced Neoplasia

**DOI:** 10.3390/ph15101273

**Published:** 2022-10-16

**Authors:** Ivan S. Mfouo-Tynga, Augustin G. Mouinga-Ondeme

**Affiliations:** Unité des Infections Rétrovirales et Pathologies Associées, Centre International de Recherche Médicales de Franceville (CIRMF), Franceville BP 769, Gabon

**Keywords:** oncogenic viruses, neoplasia, photodynamic therapy, photodynamic inactivation, nanomaterials, immunity, signaling pathways

## Abstract

The recent COVID-19 pandemic outbreak and arising complications during treatments have highlighted and demonstrated again the evolving ability of microorganisms, especially viral resistance to treatment as they develop into new and strong strains. The search for novel and effective treatments to counter the effects of ever-changing viruses is undergoing. Although it is an approved procedure for treating cancer, photodynamic therapy (PDT) was first used against bacteria and has now shown potential against viruses and certain induced diseases. PDT is a multi-stage process and uses photosensitizing molecules (PSs) that accumulate in diseased tissues and eradicates them after being light-activated in the presence of oxygen. In this review, studies describing viruses and their roles in disrupting cell regulation mechanisms and signaling pathways and facilitating tumorigenesis were described. With the development of innovative “or smart” PSs through the use of nanoparticles and two-photon excitation, among other strategies, PDT can boost immune responses, inactivate viral infections, and eradicate neoplastic cells. Visualization and monitoring of biological processes can be achieved in real-time with nanomedicines and better tissue penetration strategies. After photodynamic inactivation of viruses, signaling pathways seem to be restored but the underlying mechanisms are still to be elucidated. Light-mediated treatments are suitable to manage both oncogenic viral infections and induced neoplasia.

## 1. Introduction

### 1.1. Historical and Background of Photodynamic Therapy

Photochemical therapy involves reactions initiated by the absorption of photons. The direct outcome of such absorption is the creation of transient excited states, whose chemical and physical properties differ greatly from those of their original or ground states. The excited substances can interact with nearby molecules and act as stronger acids or reducing agents when compared to their counterpart ground states. Light-sensitive substances are essential to initiate some processes including photosynthesis, rhodopsin isomerization, vitamin D production, photography, xerography, semiconductor chips, and many others [1]. *Photodynamic* actions refer to the requirement of oxygen in the targeted microenvironment for effective photosensitization, initiating reactions through the use of agents able to facilitate light absorption coupled with energy-transferring to the desired reactants. Photodynamic therapy (PDT) is an alternate form of photochemical therapy that has been widely practiced in dermatology [2]. PDT is a multi-stage process that selectively causes photo-antiproliferative effects in cancerous cells, coupled with free radical production and action to induce apoptosis in aggressive and resistant neoplastic cells [3]. Photosensitizing substances, also called photosensitizers (PSs), are (systemically or topically) administered in the dark and preferentially accumulate in diseased structures and areas. In the presence of molecular oxygen, they effectively eradicate targeted cells after light activation and the generation of reactive oxygen species (ROS). It is known as a minimally invasive and minimally toxic treatment that offers additional advantages over conventional treatments. Other light-mediated therapies such as wound healing, rejuvenation, or hair removal do not require PS or tissue oxygen [4]. Photochemical substances have been used to treat conditions such as psoriasis and vitiligo by applying psoralen-plant extracts and light in Ancient Egypt, India, China, and Greece [4,5,6,7,8].

PDT was unintentionally discovered when Oscar Raad observed that a fluorescent product, generated by the interaction of acridine and light, was toxic to paramecium, and had antibacterial activities. Skin cancer, lupus, condyloma, and numerous cutaneous conditions have been successfully treated with eosin-mediated PDT [9]. Eosin is a dye used to stain cytoplasm, red blood cells, collagen, and muscle fibers for histological examination. Eosin fluorescence is utilized for diagnostics. It has great photodynamic potency and is currently utilized as PSs in the treatment of palmar and axillary hyperhidrosis [10]. The history of PDT is linked to the development of PSs throughout the years. Hematoporphyrin (Hp), another plant dye, and its derivatives (HpD) produced better therapeutic effects and higher biosafety levels than the first PSs, namely acridine, eosin, and chinidine. The purified or derivatives Hp (HpD) showed better efficacy for neoplastic conditions than their precursor. Photofrin was the first HpD to be reported for human skin cancer and remains the most commonly used PS worldwide. Since then, many kinds of PSs are being synthesized and tested to enhance the therapeutic efficacy of PDT over a wider range of diseases and infections [9,11]. 

### 1.2. Emerging Challenges

The efficacy of PDT depends on the physicochemical features of PSs. Due to the high reactivity and short half-life of photo-induced ^1^O_2_ and other free radicals, structures that are affected by PDT have to be close the aforementioned radicals as their half-lives last less than 40 nanoseconds [12]. Ideal PSs should have minimal dark toxicity toward all kinds of cells, light activation in the visible or near-infrared region of the spectrum, minimal light toxicity to normal cells, high yield of ROS, and effective curative effects at therapeutic doses to the diseased cells [11]. Lights in the therapeutic window (visible and near-infrared wavelengths) can excite suitable absorbing PSs. These wavelengths usually offer limited light penetration into deep-seated tissues but activate PSs on the surface or within a couple of millimeters in depth. As a result, they are unable to treat bulking tissues or those localized inside the body [9]. For optimal PS activation, and even when the depth coincides with the distance of the targeted tissues, the light dose has to be correctly determined and adjusted, and in some instances, delivered in pulses (non-continuous) [13].

ROS generation depends on the cellular oxygenation in the microenvironment of targeted tissues. Hyperactive cells rely on the vasculature for their need of nutrients and oxygen. If the supply systems are not adequate, certain diseased and/or high proliferating cells can rapidly run out of oxygen due to accelerating growth and high oxygen diffusion [14]. During PDT, the efficacy can significantly decrease as the cellular oxygen level drops. PDT is known as a self-limiting modality because it sometimes causes its inhibition by damaging the vasculature of the treated tissues [15,16]. In return, damaged oxygen supply systems promote hypoxia, which further reduces the efficacy of treatment [17]. High singlet oxygen quantum yield in the aqueous environment is an essential criterion for ROS generation [18]. The physicochemical features of PSs affect their successful accumulation and activities in the targeted tissues. One of the major issues is the solubility of PSs and aggregation in aqueous environments, leading to ineffective photodynamic actions [19]. Through chemical manipulations, both the water solubility and singlet oxygen generation can be improved [20,21,22,23,24]. Adenosine triphosphate (ATP)-binding cassette (ABC) proteins are ATP-dependent pumps that belong to a class of membrane-bound transporters. They move substrates in (influx) and out (efflux) of cells and control the movement of most drugs and metabolites across the cell surfaces and organelle membranes [24]. High expression of efflux proteins promotes the inaccessibility of PSs inside the targeted and diseased tissues. The ABC transport proteins prevent therapeutic agents from entering the desired tissues, and this constitutes a major setback, leading to the development of cancer resistance to treatment [25]. Diseases may develop resistance to any type of therapeutic approach through different mechanisms. The development of resistance to therapy is a major setback and promoter of disease progression [26]. 

Recurrence or/and refractory diseases are becoming common and mediated through drug resistance, which is more evident with limited treatment efficacy. Several factors may contribute to drug resistance and include stem cell regulation, increased DNA damage, epigenetic and microRNA regulation, drug–target mutation, and reduced cell death induction [25,27,28]. Resistance to different drugs is known as multidrug resistance (MDR), which prevents drug accumulation through ATP-dependent pumps. The ATP-dependent proteins negatively affect the uptake of xenobiotics and mediate their excretion in the bile and urine [29]. Over 2.8 million people are diagnosed with infectious diseases caused by antibiotic-resistant microorganisms in the United States each year. As these emerging forms of infections increase, the so-called superbugs are currently claiming more than 35,000 lives lost annually [30]. The occurrence of MDR and antimicrobial resistant (AMR) pathogenic microorganisms in human and veterinary medicine are causing tremendous fatalities and becoming major societal threats [31,32]. Aside from the aforementioned, various factors can impact both conventional and non-conventional treatments including PDT and limit the outcomes. Skin sensitivity is a common reaction where a patient’s skin becomes photosensitive, even to sunlight, for a few weeks. This is seen as a major drawback, while other undesirable effects could be significantly reduced by adjusting the parameters of PDT. Such improvements will give rise to the enhanced treatment of a variety of neoplastic conditions, but also in non-cancerous diseases related to ophthalmology, dermatology, cardiology, viral inactivation, and blood purification [33].

## 2. Therapeutic Improvements 

### 2.1. Emergence of “Smart” Light-Sensitive Molecules

Light-sensitive molecules (PSs) induce chemical changes in other molecules due to their ability to absorb photons and successfully pass them onto nearby molecules. To overcome certain limitations, second generations PSs were developed from HpD counterparts and other precursors. Recently, third-generation PSs have been examined for their therapeutic efficacy after also being developed based on specific features and limitations of second-generation PSs. Bioconjugation of second-generation PSs with targeting moieties and their encapsulation into carriers constitute the main route of synthesis [34]. The improvements made to the core properties of first-generation PSs have led to second-generation PSs that have better solubility and enhanced phototoxicity activity. The peripheral features are modified by conjugation or encapsulation of specific moieties, leading to the development of third-generation PSs that possess great biostability and better targeting abilities to achieve improved delivery and accumulation into the desired cells or areas. Both bioconjugation and encapsulation are strategically conducted to allow for enhanced affinity to targeted and diseased tissues. In the cancer scenario, newly developed PSs have a stronger affinity toward cancerous, cancer stem, tumor vascular endothelial cells, and other cellular structures that are highly expressed in diseased tissues [35]. Recombinant DNA technology is an interesting approach that could offer multiple possibilities and among these is the capability to produce fusion proteins made up of fluorescent PSs associated with DNA targeting domains. Such products could be utilized as theranostic agents. Another approach is photo-immunoconjugation, which uses antibodies linked to PSs, able to recognize membrane-bound receptors of targeted cells [36,37].

Nanotechnology is a term used to designate an area of science and engineering that studies events that take place at the nanoscale. It has a wide range of applications in medical treatments, pharmaceuticals, food, and the electronics industries. With innovative nanotechnology, applications of PDT in deep-seated tissues and enhanced therapy can be achieved. Nanomedicine involves the use of nanoparticles (NPs) and nanodevices in delivering drugs, monitoring conditions, and diagnosing diseases. PDT was restricted to superficial conditions, mostly skin-related illnesses, due to limited light penetration depth, low oxygen, and PS concentration in the hypoxic core or inside targeted areas. With nanomedicine, NPs serve as a hyper-class of carriers and help achieve enhanced PS accumulation, increased oxygen transport, and hypoxia relief in a tumor microenvironment (TME) [38,39]. Below a diameter of 100 nm, nanospheres or capsules mediate the transport and controlled release of hydrophobic drugs in blood, the modification of their surface areas with functional groups for more biochemical interactions, a large distribution of volume, and drug uptake by the host cells [40,41].

Numerous nanocarriers include polymers, micelles, liposomes, dendrimeric as well as inorganic structures. Novel and biocompatible nanocarriers might encapsulate and provide additional targeting effects of PSs for better anticancer actions [42]. Niosomes are advanced and better nanocarriers as they are more stable than other nanocarriers used in drug-delivery systems. One of the effects of NPs, when used in combination with PSs, is the prolonged circulation and accumulation of PSs in the TME. NP-carriers can enhance PS accumulation in tumor sites by the enhanced permeability retention (EPR) effect and specifically targeting neoplastic and endothelial cells [43]. The EPR effect depends on the leaky vasculature of solid tumors that has several pores. NPs easily recognize and enter into the targeted TME through the porous vasculature [44]. Additionally, nano-encapsulators protect and control the release of PSs, thus the pharmacokinetics of such nanoconjugates are mainly dependent on the physicochemical characteristics of NPs [45]. Through the development of nanocarrier complexes, not only is better targeting and the accumulation of therapeutic agents being achieved but also the possibility of overcoming hypoxia and metastatic outturns. Multifunctional nanoplatforms are being developed and might provide better targeting and accumulation in TME, enhanced pharmacokinetics and pharmacodynamics, and improved therapeutic outcomes [46]. 

### 2.2. Light-Related Strategies

The light penetration depth into target tissues is an important parameter in photoactivated therapy. The absorption mechanisms are complex as light can be reflected, scattered, and absorbed. Light absorption also depends on the tissue constitution of chromophores including hemoglobin, myoglobin, and cytochromes. These endogenous chromophores can enter a competition with PSs, thus reducing the possibility of light absorption and subsequent photodynamic effects. The electromagnetic spectrum range from 600 to 1200 nm is known as the tissue optical window and is mainly considered when multifunctional complexes are been developed. The phototherapeutic window (600–850 nm) is mainly targeted for proper photodynamic actions. Below, shorter wavelengths offer more absorption, which results in increased skin photosensitivity but limited tissue penetration. While better tissue penetration is achieved with longer wavelengths, it does not have sufficient energy to excite PSs and produce sufficient ROS for subsequent damage and death [47]. The NIR light at about 830 nm is seemingly the optimal region for both PS absorption and suitable tissue penetration. Good light penetration at that wavelength was reported and reached formalin-treated tissues, bone, and brain [48]. The two-photon excitation (TPE) technique is now being preferred over the commonly used activation (one-photon excitation, OPE) of PSs. Activation through TPE improves the light penetration depth in thicker tissues and samples, coupled with other beneficial effects including very limited photo-damage to normal tissues when compared to OPE, and increased imaging for diagnosis [49]. TPE is ideally used for diagnostic applications and provides 3-dimensional imaging without photo-bleaching and photo-toxicity or any other damage to the plane of focus. For in-depth analysis of live tissues or small animals, TPE is also preferred over confocal microscopy [49]. Certain NP-modified PSs have shown characteristics of luminescent materials when activated through TPE [50,51,52,53,54]. Several NP-formulated complexes have aggregation-induced emission (AIE) effects and have shown great PDT capabilities with high fluorescent quantum yield [54,55,56,57].

### 2.3. Few Supplying Oxygen Strategies

To successfully induce photodynamic effects, PDT also depends on the O_2_ supply. One of the possible ways is to alternate the photochemical structure of PSs by using antennae fullerene complexes and switching from type II to type I reactions [58]. Although the oxygen-independent type I route might maintain PDT actions, hypoxia in TME must be dealt with at some stage. Hypoxia can be overcome through several means and using PS-multifunctional complexes containing specific hypoxia probes can constitute a way out. When light-activated, the hypoxia probes in microcarriers can trigger O_2_ release, leading to enhanced PDT effects [59]. Another plausible option is to use micelles formed by PSs conjugated to bio-stable nanocarriers containing hemoglobin polymers to generate O_2_ and subsequently exert great phototoxicity [60]. Hypoxia-responsive nanocarriers are another class of intelligent molecules with ROS-generating abilities in hypoxic conditions. Some hypoxia-responsive nanocarriers are equipped with azobenzene at their core and are stabilized together with PSs in micelles, and their light-activated forms cause the continuous conversion of O_2_ to ^1^O_2_ to induce damage to neoplastic cells in type II reactions. Such micelles can incorporate several therapeutic agents yielding synergistic effects [61]. Additionally, the pulse irradiation mode is commonly used as the continuous wave mode of irradiation but provides a more efficient way of generating ^1^O_2_ due to re-oxygenation [62]. It also induces apoptosis as the main cell death program, which is a better response than the necrotic response. In pulse mode, the breaks between repeats create a high level of O_2_ in pulse irradiation, allowing the replenishment of O_2_ and tissue re-oxygenation [63,64]. The ratio between the pulse repetitions and the total pulse irradiation time is known as the intermittency index, which is directly proportional to the ROS generation and PDT response [62]

### 2.4. Mechanisms of Photodynamic Actions

**Figure 1:**
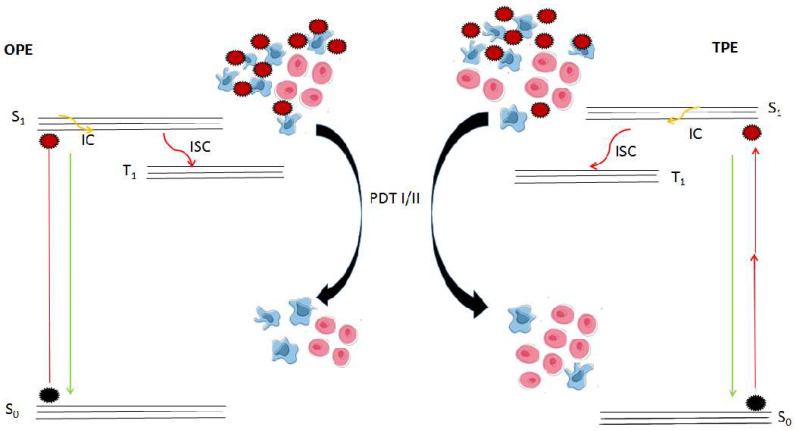
Modified Jablonski diagram. Different states of photosensitizers (PSs) from the ground state (S_0_) to the activated or excited state (S_1_) to fluorescence or conversion (IC and ISC) into the excited triplet state (T_1_). Triplet state PSs induce photodynamic reactions (PDT I/II), killing of diseased cells, and a reduction in tumor. PSs (S_0_) are excited through one (OPE) or two (TPE) photon routes. The tumor-size is reduced in both routes, but normal cell growth is being promoted with TPE.

After undergoing intersystem crossing, triplet state PSs can interact with nearby substrates for energy transfer and the formation of superoxide anion radicals and other free radicals in type I photo-reactions, or with molecular oxygen in the type II photo-reaction to give rise to the singlet oxygen (^1^O_2_). The ground state of molecular oxygen is a triplet state. The PS returns to the ground state (S_0_) from the excited state (S_1_) while promoting the formation of ^1^O_2_. In type II photo-reaction scenarios, the cycle can be repeated and PSs can also be excited, undergoing the same processes and generating more ^1^O_2_. Eventually, both types generate ROS, which are toxic agents leading to oxidative damage and subsequent cell death. Figure 1 depicts the different states or forms of PSs from the ground state (S_0_) to induce photodynamic reactions (PDT I/II) and the killing of neoplastic cells. Activation or excitation can be conducted through one-photon or two-photon excitation (OPE and TPE) routes. Ground state PSs are being excited by light, which enables the transition into the singlet excited state (S_1_). In both routes, the tumor size was significantly reduced, but normal growth and conditions are being restored and promoted with TPE. Restoring events are promoted after light exposure to normal tissues as light alone is known to stimulate cell growth in the wound healing process.

However, type II usually predominates over type I reactions; the level of molecular oxygen determines the occurrence of ^1^O_2_ and photodynamic reactions in TME [55,56]. Therefore, strategies that maintain sufficient oxygen levels in TME are being developed to ensure limited hypoxia and therapeutic efficacy. Strategies might include metal-organic framework (MOF) nanomaterials, fluorine-mediated nanocarriers, or hydrogen peroxide (H_2_O_2_) endogenous decomposition. Biomimetic nanoplatforms containing MOF store up oxygen and later release NIR light-responsive oxygen inside cancer cells during PDT, as reported by Gao et al. [57]. Fluorinated-nanocarrier conjugates could effectively induce phototoxicity after delivering oxygen inside cancer and hypoxic cells [63,64,65]. 

Furthermore, certain nanocarriers with catalytic activities can mediate chemical decomposition reactions that increase oxygen, leading to the subsequent reduction of hypoxia in TME. Manganese oxide (MnO_2_) is often considered as a hypoxia reliever, it can react with H_2_O_2_ and is decomposed into Mn^2+^ and oxygen under an acidic microenvironment. Multifunctional NP complexes containing MnO_2_ can trigger the decomposition of H_2_O_2_ into oxygen as the main byproduct during PDT. Facilitated by magnetic field attractions, MnO_2_-nanocarrier mediated-PDT led to increased concentrations of PSs and oxygen inside hypoxic cells [66,67,68,69]. The integration of MnO_2_ into NP-based platforms helps in sustaining sufficient levels of oxygen inside cancer cells and the magnetic-induced accumulation of PSs [70,71]. Carbon-dots (CDs) have been identified as highly efficient singlet oxygen promoters; in association with MnO_2_, they formed an impressive H_2_O_2_-driven oxygenator that boosted the PDT effects on solid hypoxic tumors in an acidic microenvironment [72].

## 3. Viral Infections and Photodynamic-Induced Immunity

### 3.1. Induced Immunity

Humans have various mechanisms that work together to provide immune defense and prevent viral infections. Immune responses result from a series of events triggered by foreign invaders including viruses, bacteria, fungi, prions, or other microbes. An immune reaction involves defense mechanisms against harmful invaders and is initiated by recognizing their antigens. Antigens are usually proteins on the surfaces of invaders, not limited to living substances, and can also be toxins, chemical drugs, or any foreign particles. We could distinguish three main classes of immunity: against all antigens or innate immunity; against various antigens developed especially after exposure or acquired immunity, and passive immunity, which is targeted against specific antigens as a result of exposure to antibodies, immunization, or placental substances [73]. An inflammatory response often occurs after tissues are injured or invaded, causing the release of chemicals, which induce swelling or inflammation. PDT-induced destruction of tumor tissues also leads to immediate and localized inflammatory responses that assist in containing and removing debris. Preclinical and clinical studies have reported that PDT induced innate and adaptive immune responses as well as the release of pro-inflammatory proteins. PDT-induced immune responses tend to increase the beneficial effects of the anticancer or antibacterial treatment [74]. The resulting death or immunogenic cell death (ICD) is characterized and mediated by the activities of damage-associated molecular patterns (DAMPs) [75,76]. The generation of an anti-cancer vaccine with PDT has been demonstrated whereas neutrophils have a pivotal role in enhancing anticancer treatment and suitable immune response against basal cell carcinoma in patients [74]. Additionally, such ideal therapies should stimulate the immune responses to simultaneously recognize, pursue, and eradicate both primary and emerging conditions. For successful PDT, the cellular and nuclear membranes of infected host cells could constitute a suitable target. However, most conventional cancer treatments kill any residual malignant cells at or near the primary site as well as distant metastases, but also suppress immune responses. At their efficient doses to destroy tumors, current anticancer treatments cause damage to the bone marrow, from where all cells of the immune system originate.

Viruses only exist to replicate and rely on plant, animal, or bacterial cells. First, they must penetrate and then utilize the machinery of the host cells for their replication of genetic materials. They consist of nucleic acids (DNA or RNA) at the core, protective proteins (or capsids), and optionally outer spikey coats (or envelopes). Enveloped and non-enveloped/naked (RNA/DNA) viruses are classified according to their genetic materials and reproductive modes. Most viral sizes range from 0.02 to 0.3 μm on average, but larger viruses can be up to 1 μm in diameter. After infections, some viral-induced diseases are harmless such as the common cold, diarrhea, and gastroenteritis, while others cause far more serious illnesses including human immunodeficiency virus infection and acquired immune deficiency syndrome (HIV/AIDS), Ebola, severe acute respiratory syndrome (SARS) or Zika infections [77,78]. The plausible PDT-induced mechanisms may affect the infected cells as well as the target viruses. The nuclear materials of viruses can be directly targeted by UV-c irradiation. Both UV-c and PS-mediated PDI could destroy viral genetic materials and the spike proteins, but UV-c will specifically damage nuclear material, while PSs-mediated PDI could mostly destroy the spike and viral membrane. Together, they lead to limited viral entry into the cells, the suppression of viral replication, and significant viral inactivation. The PSs used would maximally be localized in diseased/infected cells, leaving normal cells unaffected or with limited effect. Additionally, light-mediated effects should not only suppress viral infection but also prevent tumorigenesis. A recent study reported that a light-mediated therapeutic approach was able to both induce damage to the viral genome and disinfection through UV-c irradiation, and also cause damage to the viral spike and membrane, leading to no interaction with the host membrane receptors, and subsequently no transcription of the reporter protein after photodithazine-mediated PDT. Other advantages were reported including high repeatability of the results without viral resistance, and a fast removal of viruses in a very short time [79]. 

### 3.2. Oncogenic Viruses

Certain groups of viruses have cancer-inducing capabilities and are known as oncogenic viruses. The best known oncogenic viruses include Epstein–Barr virus (EBV), hepatitis B virus (HBV), human T-Lymphotropic virus-type 1 (HTLV-1), human papilloma virus (HPV), hepatitis C virus (HCV), Kaposi sarcoma (Human) herpes virus (KSHV or HHV-8) and Merkel cell polyomavirus (MCV). They induce precancerous conditions, which may or may not develop into malignancies [80]. Table 1 presents the main PS-mediated PDT/PDI that have been used and the characteristics of oncogenic viruses. Approximately, DNA oncogenic viruses including HBV, MCV, and HPV account for about a quarter of all human cancers.

Facilitated by immune-deficiency conditions, HPV infection is one of the most common oncogenic infections and is implicated in more than 50% of all infections associated with cervical cancer in females. The virus genome is a linear double-stranded DNA (7–8 Kbp) and is mainly transmitted by mucosal contact, skin-to-skin contact, microabrasion in the skin, or mucosal epidermal surface. Both HPV-16 and 18 are forms directly related to cervical cancer [83,98]. The human herpesvirus-4 (HHV-4) or EBV was the first oncogenic virus to be discovered and its genome is a linear double-stranded DNA (168–184 Kbp). EBV is lymphotropic but also infects epithelial cells that are the primary site of replication [84,105]. The HTLV-1 infection is an important factor in the carcinogenesis of T-cell leukemia [91,98]. Both HTLV-1 and HCV have RNA genomes, with the latter belonging to the flavivirus with a positive polarity. Generally, HCV infections are asymptomatic but are the main causes of liver transplantation in the USA and the Western world, and can lead to hepatocellular carcinoma, liver damage, and cirrhosis. It is sexually or intravenously transmitted and spreads well with more than 200 million infected people worldwide and a high prevalence among prison inmates [89,106]. Along with their hepatitis counterpart, they often cause chronic inflammation. Mostly reported in Sub-Saharan Africa and East Asia, HBV infections cause cirrhosis, acute liver infection, and failure, which requires transplantation. Related to the development of hepatocellular carcinoma, the circular and double-stranded DNA hepadnavirus member is transmitted via blood and body fluids. More than 50% of infections are asymptomatic and require an incubation of 3 months at least before the first symptoms are manifested [96,97]. The gammaherpesvirus (KSHV/HHV-8) exists in its latent form but when the episome that expresses the latency-associated nuclear antigen (LANA) is activated, it can replicate to yield linear DNA of around 140 Kbp. The variable rate of reproduction does not affect the ability to infect B-lymphocytes, endothelial cells, macrophages, and keratinocytes. HHV-8 causes a rare skin tumor, Kaposi’s sarcoma, and other diseases including effusion lymphoma and multicentric Castleman’s disease, all after the effects of HIV/AIDS are evident [81,87]. The last to be discovered, MCV is a 5.4 Kbp-size circular DNA genome that easily integrates the cell genome. MCV infection usually occurs under immunosuppression and leads to Merkel cell carcinoma, a rare but aggressive malignancy [106].

## 4. Oncogenic Viruses—Signaling Pathway Interactions

Oncogenic viruses are believed to play a major role in cell transformation and tumorigenesis. Such mediated transformations are brought about through alternations of molecular mechanisms related to cell cycle, proliferation, survival, and death. Disrupted mechanisms induce unregulated cell cycle, uncontrolled proliferation, and facilitated conversion into cancer forms by pro-oncogenic effectors. As viral infections progress, viruses can evolve and develop mutations, tumors, or resistance (to the treatment of immune defense). When one or more diseases emerge as the result of a primary disease, it is known as co-morbidity, the coexistence of two or more diseases in a patient. Most sexually transmitted infections spread to other parts or systems of the body. In the case of co-morbidity that includes systemic infection, the resulting condition becomes a complex issue and requires an appropriate therapy that should neutralize both the primary and emerging diseases in patients with limited if no impact on immune response [84,85].

Infections with oncogenic viruses affect and cause the diversion of conserved signaling pathways in favor of carcinogenesis. The phosphatidylinositol 3-kinases (PI3K)-Akt-mechanistic target of rapamycin (mTOR, PI3K-Akt-mTOR) is a cascade signaling pathway, which is often disrupted by viral infection [107]. Activated PI3K mediates Akt activation, which in return further activates downstream effectors through phosphorylation including mTOR [108,109]. mTOR ensures the proper phosphorylation and translation of proteins such as the 4eBP1 initiator that control cell proliferation [109]. Disruption in the oxygen-dependent crosstalk PI3K-Akt-mTOR signaling pathway causes the alternation of the 4eBP1 initiator’s functions and cell proliferation. Under hypoxic conditions, the suppression of senescence can occur as a result of synergistic effects. Senescence involves the inhibition of cell proliferation through cell cycle arrest [110,111]. Another cascade signaling pathway that is often disturbed by oncogenic viruses is the mitogen-activated protein kinase (MAPK), which is activated by stimuli such as growth factors, heat shock, and osmotic or oxidative stress. Normal MAPK is involved in tumor suppression and antimicrobial activities, through the induction of senescence. Generally, mutations lead to upregulation and hyperactivity of kinases operating in MAPK. This high activity of MAPK is unable to suppress tumorigenesis [112,113]. 

Affected also by oncogenic infections, the Notch signaling pathway functions as a cell regulator for cell monitoring of mobility, differentiation, proliferation, and fate. Additionally, this signaling has both protumorigenic and suppressing effects, and its oncogenic activity was first related to the pathogenesis of T-cell acute lymphoblastic leukemia/lymphoma (T-ALL), and then colon cancer, lung carcinoma, and Kaposi’s sarcoma [114]. Although HPV infection is unable to induce cancer alone, the persistent infection has been revealed to have a stronger impact, and HPV is known as the primary causal factor for cervical carcinogenesis [115,116]. The imbalance in structural and β-catenin promotes the occurrence of various cancers including cervical cancer. The WNT/β-catenin signaling pathway activates the cell surface receptors and stabilizes B-catenin for the regulation of stem cell renewal, maturation, and proliferation [115]. Human oncogenic viruses including HTLV-1, HHV-8, and EBV can have prolonged effects on cytoplasmic nuclear factor-kB (NF-kB), a group of transcriptional factors that co-exist in complex with their inhibitors, IkB proteins. Usual activation is rapid and ensures the induction of target genes involved in inflammation, immunity, cell proliferation, or death. After gene expression, IkB proteins bind back to NF-kB, leading to the deactivation of IkB kinases to restore the sequestration state of the NF-kB signaling pathway [116,117,118]. All viral infections invade and alter the nuclear activities and materials of their hosts, causing damage, dysfunction, and/or diseases. If the nuclear damage response is not effective, it gives rise to the possible mutation and development of resistance to therapy.

## 5. Future Perspective and Conclusions

During replication, several viruses can cause mutations, and some mutations lead to carcinogenesis. Most oncogenic events are usually preceded by immune suppression, which is caused by other pathogens such as HIV/AIDS for HTLV-1 or HHV-8. The emergence of a precancerous state creates comorbidity, a coexistence of at least two diseases that coexist simultaneously. When designing a treatment regimen, considerations must be made to effectively deal with each existing condition or pathology. Most anticancer treatments (chemotherapy and radiotherapy) further suppress the immune system by damaging the bone marrow and other immune components as part of their unspecific side effects. Effective treatments, in such circumstances, should heal the different pathologies with limited damage to normal and healthy structures. Thus, a multi-targeted approach is an option that should be applied when considering treatment for virus-induced cancer. PDT is widely recognized as a treatment for non-melanoma skin and is often considered as an anticancer therapy due to its high selectivity for neoplastic tissues. It has antimicrobial effects and constitutes a novel strategy. The PDT-mediated antiviral effects not only inactivate infections, but also seem to reinstate immune mechanisms, which are generally suppressed by traditional treatments.

PSs are the pivotal elements in all PDT as they are designed to target or interact with multiple entities including cellular components from various tissues, light radiation from different wavelengths (range), molecular oxygen, etc. The success of PDT mainly depends on the physicochemical features, hence they are constantly being improved. The therapeutic improvement is directly proportional to the structural improvements made to PSs. Nanotechnology offers tremendous opportunities and helps to improve targeting strategies, leading to enhanced therapeutic outcomes. In some other cases, it allows for better conditioning for therapeutic actions, as discussed with hypoxia relievers. However, biocompatible (or organic) nanoparticles must be prioritized and considered over others. Many studies, both in vitro and in vivo, do not specify the fate of the used nanoparticles after PDT, nor the clearance mechanisms associated with them. More is still to be done to clarify these important biosafety issues. Alternatively, herbal nanomaterials could be considered for conjugation and multicomplex formations with PSs. Many medicinal drugs are developed from plants, and the synthesis of PSs derived from plant extracts should be encouraged. Both herbal derivatives hypericin and curcumin-based PSs work well in light-mediated treatments (PDT and PDI) and should be safer than other synthetic PSs as the green approach in PDT generally leads to low systemic cytotoxicity.

Finally, multifactorial PDT is efficient in effectively treating comorbidity (viral infection and oncogenic cancer) and triggering antitumor immune responses. This flexible modality can be enhanced through recombination and conjugation methods to more specifically target viral DNA and capsids. Through TPE activation, the tumor size is significantly reduced while normal cell growth and regulatory mechanisms are being promoted and restored. This restoring role is well-known and observed in wound healing when light alone has a stimulating effect on the cell growth, proliferation, and signaling pathways. TPE-activated treatments can not only induce viral inactivation (PDI) and tumor damage and also decrease normal cell growth (PDT) and biological event visualization and monitoring in real-time (Photodiagnosis, PDD). The utilization of TPE enhances the depth of light-penetration into tissues, rendering light-based treatment (PDT/PDI/PDD) applicable to embodied conditions and no longer restricted to superficial ones. PDT has a restoring/stimulating effect on the immune system; if similar effects can be observed and verified in signaling pathways and cell control mechanisms, it would make PDT even more appealing. 

## Figures and Tables

**Table 1:** The PS-mediated PDT/PDI and characteristics of oncogenic viruses.

PSs	Tropisms	Cancer	Primary Infection	Viral Integration	Virus	Family	Virus Type	Transmission	Refs.
5-ALA mTHPc Curcumin	Keratinocytes, stratified epithelial cells	Cervical carcinoma, pharyngeal carcinoma, anal carcinoma, penile carcinoma	Warts, condyloma, acumatum, oral and laryngeal papillomatosis	Yes	HPV-16 HPV-18	*Papillomavirus* *Viridae*	Enveloped DNA	Sexual contact, Mucosal contact	[80,81,82,83,84,85,86]
PBNP Zn-Bc_Am	B-lymphocytes, epithelial cells	Burkitt’s lymphoma, nasopharyngeal carcinoma	Asymptomatic, mononucleosis	Yes	ERV/ HH-4	*Herpesvirus* *Viridae*	Enveloped DNA	Saliva	[84,85,86,87]
Photosens Chlorin e6	Macrophages, keratinocytes, endothelial cells, B cells, etc.	Kaposi’s sarcoma, effusion lymphoma, multicentric Castleman’s disease	Asymptomatic	Yes	KSHV/ HHV-8	*Herpesvirus* *Viridae*	Enveloped DNA	Sexual contact	[84,85,86,88,89,90]
Curcumin, Methylene Blue	Hepatocytes	Hepatocellular carcinoma	Acute hepatitis and chronic (10%)	Yes	HBV	*Hepadnavirus* *Viridae*	Enveloped DNA	Sexual contact, parental	[84,85,86,89,91,92,93]
Curcumin, Methylene Blue	Hepatocytes, B-lymphocytes, dendritic cells	Hepatocellular carcinoma	Acute hepatitis and chronic (85%)	No	HCV	*Flavivirus* *Viridae*	Enveloped RNA	Sexual contact, parental	[84,85,86,94,95,96]
5-ALA Hypericin	T-lymphocytes	Adult T-cell leukemia	Asymptomatic	Yes	HTLV-1	*Retrovirus* *Viridae*	Enveloped RNA	Fluids with cells	[84,85,86,97,98,99,100,101]
Quinacrine	Merkel’s cells	Merkel cell carcinoma	Asymptomatic	Yes	MCV	*Polymavirus* *Viridae*	Naked DNA	Not clear (respiratory droplets)	[84,85,86,102,103,104]

## Data Availability

Data sharing is not applicable to this article.

## Abbreviations

ABC	ATP-binding cassette	Kbp	Kilo base pair(s)
AIDS	Acquired immune deficiency syndrome	KSHV	Kaposi’s sarcoma herpes virus
AIE	Aggregation induced emission	LANA	Latent associated nuclear antigen
AMR	Antimicrobial resistance	MAPK	Mitogen-activated protein kinase
ATM	Ataxia-telangiectasia mutated	MCV	Merkel cell polymavirus
ATP	Adenosine triphosphate	MDR	Multiple drug resistance
ATR	Ataxia-telangiectasia Rad3	MOF	Metal organic framework
ART	Antiretroviral treatment	MnO_2_	Manganese oxide
CD	Carbon dots	mTOR	Mechanistic target of rapamycin
CHK	Checkpoint kinase	NF-kB	Nuclear factor kB
DAMPs	Damage associated molecular patterns	NIR	Near infrared
DNA	Deoxyribonucleic acid	NPs	Nanoparticles
EBV	Epstein–Barr Virus	^1^O_2_	Singlet oxygen
EPR	Enhanced permeability retention	OPE	One photon emission
FG	Fluorescence-guided	PDD	Photodynamic diagnosis
GBM	Glioblastoma	PDI	Photodynamic inactivation
HBV	Hepatitis B virus	PDT	Photodynamic therapy
HCV	Hepatitis C virus	PI3K	Phosphatidylinositol-3-kinase
HHV	Human herpes virus	PSs	Photosensitizers
HIV	Human immune virus	RNA	Ribonucleic acid
Hp	Hematoporphyrin	ROS	Reactive oxygen species
HpD	Hematoporphyrin derivatives	SARS	Severe acute respiratory syndrome
HTLV	Human T-lymphotropic virus	S_0_/S_1_	Ground state/excited state
H_2_O_2_	Hydrogen peroxide	T-ALL	T-cell acute lymphoblastic leukemia
IC	Internal conversion	TPE	Two photon emission
ICD	Immunogenic cell death	TME	Tumor microenvironment
ISC	Intersystem crossing	T_1_	Excited triplet state

## References

[1] Krueger B.P., Fleming G.R., Longworth J. (2018). Photochemical reaction. Ency. Brit..

[2] Dougherty T.J. (1998). Photodynamic Therapy. JNCI.

[3] Barreca M., Ingarra A.M., Raimondi M.V., Spanò V., de Franco M., Menilli L., Gandin V., Miolo G., Barraja P., Montalbano A. (2022). Insight on pyrimido[5,4-g]indolizine and pyrimido[4,5-c]pyrrolo[1,2-a]azepine systems as promising photosensitizers on malignant cells. Eur. J. Med. Chem..

[4] Josefsen L.B., Boyle R.W. (2008). Photodynamic Therapy and the Development of Metal-Based Photosensitisers. Met. Based Drugs.

[5] Wilson B.T., Mang T. (1995). Photodynamic therapy for cutaneous malignancies. Clin. Dermatol..

[6] Ackroyd R., Kelty C., Brown N., Reed M. (2001). The History of Photodetection and Photodynamic Therapy. Photochem. Photobiol..

[7] Daniel M.D., Hill J.S. (1991). A history of photodynamic therapy. Aust. N. Z. J. Surg..

[8] Lee C.N., Hsu R., Chen H., Wong T.W. (2020). Daylight photodynamic therapy: An update. Molecules.

[9] Li W.P., Yen C.J., Wu B.S. (2021). Wong TW. Recent Advances in Photodynamic Therapy for Deep-Seated Tumors with the Aid of Nanomedicine. Biomedicine.

[10] Fadeel D.A.A., Fadel M., Tawfik A., Omar Y. (2022). Transfersomal eosin topical delivery assisted by fractional CO_2_ laser for photodynamic treatment of palmar hyperhidrosis: Case study. Drug Deliv. Transl. Res..

[11] Szeimies R.M., Dräger J., Abels C., Landthaler M., Calzavara-Pinton E.-G., Szeimies R.-M., Ortel B. (2001). History of photodynamic therapy in dermatology. Photodynamic Therapy and Fluorescence Diagnosis in Dermatology.

[12] Moan J., Berg K. (1991). The photodegradation of porphyrins in cells can be used to estimate the lifetime of singlet oxygen. Photochem. Photobiol..

[13] Xiao Z., Halls S., Dickey D., Tulip J., Moore R.B. (2007). Fractionated versus Standard Continuous Light Delivery in Interstitial Photodynamic Therapy of dunning Prostate Carcinomas. Clin. Cancer Res..

[14] Vaupel P., Thews O., Hoeckel M. (2001). Treatment Resistance of Solid Tumors. Med. Oncol..

[15] Henderson B.W., Waldow S.M., Mang T.S., Potter W.R., Malone P.B., Dougherty T.J. (1985). Tumor Destruction and Kinetics of Tumor Cell Death in Two Experimental Mouse Tumors Following Photodynamic Therapy. Cancer Res..

[16] Dolmans D.E., Kadambi A., Hill J.S., Waters C.A., Robinson B.C., Walker J.P., Fukumura D., Jain R.K. (2002). Vascular Accumulation of a Novel Photosensitizer, MV6401, Causes Selective Thrombosis in Tumor Vessels after Photodynamic Therapy. Cancer Res..

[17] Busch T.M., Wileyto E.P., Emanuele M.J., del Piero F., Marconato L., Glatstein E., Koch C.J. (2002). Photodynamic Therapy Creates Fluence Rate-dependent Gradients in the Intratumoral Spatial Distribution of Oxygen. Cancer Res..

[18] Ossola R., Jönsson O.M., Moor K., McNeill K. (2001). Singlet Oxygen Quantum Yields in Environmental Waters. Chem. Rev..

[19] Li Y., Wang J., Zhang X., Guo W., Li F., Yu M., Kong X., Wu W., Hong Z. (2015). Highly Water-Soluble and Tumor-Targeted Photosensitizers for Photodynamic Therapy. Org. Biomol. Chem..

[20] Dubuc C., Langlois R., Bénard F., Cauchon N., Klarskov K., Tone P., van Lier J.E. (2008). Targeting Gastrin-Releasing Peptide Receptors of Prostate Cancer Cells for Photodynamic Therapy with a Phthalocyanine-Bombesin Conjugate. Bioorg. Med. Chem. Lett..

[21] Sekhosana K.E., Nyokong T. (2014). Synthesis of Ytterbium Bisphthalocyanines: Photophysicochemical Properties and Nonlinear Absorption Behavior. Opt. Mater..

[22] Rossetti F.C., Lopes L.B., Carollo A.R.H., Thomazini J.A., Tedesco A.C., Bentley M.V.L.B. (2011). A Delivery System to Avoid Self-Aggregation and to Improve In Vitro and In Vivo Skin Delivery of a Phthalocyanine Derivative Used in the Photodynamic Therapy. J. Control Release.

[23] Kuruppuarachchi M., Savoie H., Lowry A., Alonso C., Boyle R.W. (2011). Polyacrylamide Nanoparticles as a Delivery System in Photodynamic Therapy. Mol. Pharm..

[24] Vasiliou V., Vasiliou K., Nebert D.W. (2009). Human ATP-binding cassette (ABC) transporter family. Hum. Genom..

[25] Mansoori B., Mohammadi A., Davudian S., Shirjang S., Baradaran B. (2017). The different mechanisms of cancer drug resistance: A brief review. Adv. Pharm. Bull..

[26] Aniogo E.C., George B.P.A., Abrahamse H. (2019). The role of photodynamic therapy on multidrug resistant breast cancer. Cancer Cell Int..

[27] Lucena S., Salazar N., Gracia-Cazaña T., Zamarrón A., González S., Juarranz Á., Gilaberte Y. (2015). Combined Treatments with Photodynamic Therapy for Non-Melanoma Skin Cancer. Int. J. Mol. Sci..

[28] Cree I.A., Charlton P. (2017). Molecular chess? Hallmarks of anticancer drug resistance. BMC Cancer.

[29] Ambudkar S.V., Kimchi-Sarfaty C., Sauna Z.E., Gottesman M.M. (2003). *P*-glycoprotein: From genomics to mechanism. Oncogene.

[30] CDC (2019). Antibiotic Resistance Threats in the United States, 2019.

[31] Rossolini G.M., Mantengoli E. (2008). Antimicrobial resistance in Europe and its potential impact on empirical therapy. Clin. Microbiol. Infect..

[32] Muehler D., Rupp C.M., Keceli S., Brochhausen C., Siegmund H., Maisch T., Hiller K.A., Buchalla W., Cieplik F. (2020). Insights into Mechanisms of Antimicrobial Photodynamic Action Toward Biofilms Using Phenalen-1-One Derivatives as Photosensitizers. Front. Microbiol..

[33] Sibata C.H., Colussi V.C., Oleinick N.L., Kinsella T.J. (2000). Photodynamic Therapy: A New Concept in Medical Treatment. Braz. J. Med. Biol..

[34] Mfouo-Tynga I.S., Dias L.D., Inanda N.M., Kurachi C. (2021). Features of Third Generation Photosensitizers Used in Anticancer Photodynamic Therapy: Review. Photodiagnosis Photodyn. Ther..

[35] Gomez S., Tsung A., Hu Z. (2020). Current Targets and Bioconjugation Strategies in Photodynamic Diagnosis and Therapy of Cancer. Molecules.

[36] Fernandes S.R.G., Fernandes R., Sarmento B., Pereira P.M.R., Tome J.P.C. (2019). Photoimmunoconjugates: Novel Synthetic strategies to Targeted and Treat Cancer by Photodynamic Therapy. Org. Biomol. Chem..

[37] Sandland J., Boyle R.W. (2019). Photosensitizer Antibody-Drug Conjugates: Past, Present and Future. Bioconjug. Chem..

[38] Soares S., Sousa J., Pais A., Vitorino C. (2018). Nanomedicine: Principles, Properties, and Regulatory Issues. Front. Chem..

[39] Debele T.A., Yeh C.F., Su W.P. (2020). Cancer Immunotherapy and Application of Nanoparticles in Cancers Immunotherapy as the Delivery of Immunotherapeutic Agents and as the Immunomodulators. Cancers.

[40] Algorri J.F., Ochoa M., Roldan-Varona P., Rodriguez-Cobo L., Lopez-Higuera J.M. (2021). Photodynamic Therapy: A Compendium of Latest Reviews. Cancers.

[41] Mesquita M.Q., Dias C.J., Gamelas S., Fardilha M., Neves M.G.P.M.S., Faustino M.A.F. (2018). An Insight on the Role of Photosensitizer Nanocarriers for Photodynamic Therapy. An. Acad. Bras. Cienc..

[42] Lu K.Y., Li R., Hsu C.H., Lin C.W., Chou S.C., Tsai M.L., Mi F.L. (2017). Development of a new type of multifunctional fucoidan-based nanoparticles for anticancer drug delivery. Carbohydr. Polym..

[43] Zhen Z., Tang W., Chuang Y.J., Todd T., Zhang W., Lin X., Niu G., Liu G., Wang L., Pan Z. (2014). Tumor vasculature targeted photodynamic therapy for enhanced delivery of nanoparticles. ACS Nano.

[44] Kobayashi H., Watanabe R., Choyke P.L. (2013). Improving conventional enhanced permeability and retention (EPR) effects; What is the appropriate target?. Theranostics.

[45] Zamboni W.C. (2008). Concept and clinical evaluation of carrier-mediated anticancer agents. Oncologist.

[46] Park J., Lee Y.K., Park I.K., Hwang S.R. (2021). Current Limitations and Recent Progress in Nanomedicine for Clinically Available Photodynamic Therapy. Biomedicines.

[47] Yoon I., Li J.Z., Shim Y.K. (2013). Advance in photosensitizers and light delivery for photodynamic therapy. Clin. Endosc..

[48] Jared R.J., Lauren E.A., Neil I.B., Daniel M.S. (2012). Transcranial red and near infrared light transmission in a cadaveric model. PLoS ONE.

[49] Benning R.K.P., Piston D.W. (2014). Two-Photons Excitation Microscopy for the Study of Living Cells and Tissues. Curr. Protoc. Cell Biol..

[50] Zhu C., Kwok R.T.K., Lam J.W.Y., Tang B.Z. (2018). Aggregation-induced emission: A trailblazing journey to the field of biomedicine. ACS Appl. Bio Mater..

[51] Sun X., Zebibula A., Dong X., Zhang G., Zhang D., Qian J., He S. (2018). Aggregation-induced emission nanoparticles encapsulated with pegylated nano graphene oxide and their applications in two-photon fluorescence bioimaging and photodynamic therapy in vitro and in vivo. ACS Appl. Mater. Interfaces.

[52] Zhuang W., Yang L., Ma B., Kong Q., Li G., Wang Y., Tang B.Z. (2019). Multifunctional two-photon aie luminogens for highly mitochondria-specific bioimaging and efficient photodynamic therapy. ACS Appl. Mater. Interfaces.

[53] Wang S., Wu W., Manghnani P., Xu S., Wang Y., Goh C.C., Ng L.G., Liu B. (2019). Polymerization-enhanced two-photon photosensitization for precise photodynamic therapy. ACS Nano.

[54] Ho-Wu R., Yau S.H., Goodson T. (2017). III Efficient singlet oxygen generation in metal nanoclusters for two-photon photodynamic therapy applications. J. Phys. Chem. B.

[55] McLean A., Wang R., Huo Y., Cooke A., Hopkins T., Potter N., Li Q., Isaac J., Haidar J., Jin R. (2020). Synthesis and optical properties of two-photon-absorbing au25 (captopril) 18-embedded polyacrylamide nanoparticles for cancer therapy. ACS Appl. Nano. Mater..

[56] Fitzgerald F. (2017). Photodynamic Therapy (PDT), Principles, Mechanisms and Applications.

[57] Overchuk M., Zheng G. (2018). Overcoming obstacles in the tumor microenvironment: Recent advancements in nanoparticle delivery for cancer theranostics. Biomaterials.

[58] Li Q., Huang C., Liu L., Hu R., Qu J. (2018). Enhancing Type I Photochemistry in Photodynamic Therapy Under Near Infrared Light by Using Antennae–Fullerene Complexes. Cytom. Part A.

[59] Wang P.Y., Li X.M., Yao C., Wang W.X., Zhao M.Y., El -Toni A.M., Zhang F. (2017). Orthogonal near-infrared upconversion co-regulated site-specific O_2_ delivery and photodynamic therapy for hypoxia tumor by using red blood cell microcarriers. Biomaterials.

[60] Xu X., Cui Y.C., Bu H.X., Chen J.M., Li Y., Tang G.P., Wang L.Q. (2018). A photosensitizer loaded hemoglobin-polymer conjugate as a nanocarrier for enhanced photodynamic therapy. J. Mater. Chem. B.

[61] Xu Z., Pan C., Yuan W. (2020). Light-enhanced hypoxia-responsive and azobenzene cleavage-triggered size-shrinkable micelles for synergistic photodynamic therapy and chemotherapy. Biomater. Sci..

[62] Klimenko V.V., Knyazev N.A., Moiseenko F.V., Rusanov A.A., Bogdanov A.A., Dubina M.V. (2016). Pulse mode of laser photodynamic treatment induced cell apoptosis. Photodiagnosis Photodyn. Ther..

[63] Ma S., Zhou J., Zhang Y., Yang B., He Y., Tian C., Xu X., Gu Z. (2019). An oxygen self-sufficient fluorinated nanoplatform for relieved tumor hypoxia and enhanced photodynamic therapy of cancers. ACS Appl. Mater. Interfaces.

[64] Hu H., Yan X., Wang H., Tanaka J., Wang M., You W., Li Z. (2019). Perfluorocarbon-based o 2 nanocarrier for efficient photodynamic therapy. J. Mater. Chem. B.

[65] Hu D., Zhong L., Wang M., Li H., Qu Y., Liu Q., Han R., Yuan L., Shi K., Peng J. (2019). Perfluorocarbon-loaded and redox-activatable photosensitizing agent with oxygen supply for enhancement of fluorescence/photoacoustic imaging guided tumor photodynamic therapy. ACS Appl. Bio-Mater..

[66] Liu P., Xie X., Shi X., Peng Y., Ding J., Zhou W. (2019). Oxygen-self-supplying and hif-1α-inhibiting core–shell nanosystem for hypoxia-resistant photodynamic therapy. ACS Appl. Mater. Interfaces.

[67] Hai L., Zhang A., Wu X., Cheng H., He D., Wang T., He X., Wang K. (2019). Liposome-stabilized black phosphorus for photothermal drug delivery and oxygen self-enriched photodynamic therapy. ACS Appl. Nano Mater..

[68] Phua S.Z.F., Yang G., Lim W.Q., Verma A., Chen H., Thanabalu T., Zhao Y. (2019). Catalase-integrated hyaluronic acid as nanocarriers for enhanced photodynamic therapy in solid tumor. ACS Nano.

[69] Gao Z., Li Y., Zhang Y., Cheng K., An P., Chen F., Chen J., You C., Zhu Q., Sun B. (2019). Biomimetic platinum nanozyme immobilized on 2d metal–organic frameworks for mitochondrion-targeting and oxygen self-supply photodynamic therapy. ACS Appl. Mater. Interfaces.

[70] Liang R., Liu L., He H., Chen Z., Han Z., Luo Z., Wu Z., Zheng M., Ma Y., Cai L. (2018). Oxygen-boosted immunogenic photodynamic therapy with gold nanocages@ manganese dioxide to inhibit tumor growth and metastases. Biomaterials.

[71] Feng Y., Ding D., Sun W., Qiu Y., Luo L., Shi T., Meng S., Chen X., Chen H. (2019). Magnetic manganese oxide sweetgum-ball nanospheres with large mesopores regulate tumor microenvironments for enhanced tumor nanotheranostics. ACS Appl. Mater. Interfaces.

[72] Jia Q., Ge J., Liu W., Zheng X., Chen S., Wen Y., Zhang H., Wang P. (2018). A magnetofluorescent carbon dot assembly as an acidic H_2_O_2_-driven oxygenerator to regulate tumor hypoxia for simultaneous bimodal imaging and enhanced photodynamic therapy. Adv. Mater..

[73] Chaplin D.D. (2010). Overview of the immune response. J Allergy Clin. Immunol..

[74] Reginato E., Wolf P., Hamblin M.R. (2014). Immune response after photodynamic therapy increases anti-cancer and anti-bacterial effects. World J. Immunol..

[75] Reginato E., Lindenmann J., Langner C., Schweintzger N., Bambach I., Smolle-Juttner F., Wolf P. (2014). Photodynamic therapy downregulates the function of regulatory T cells in patients with esophageal squamous cell carcinoma. Photochem. Photobiol. Sci..

[76] Wachowska M., Muchowicz A., Demkow U. (2015). Immunological aspects of antitumor photodynamic therapy outcome. Central-Eur. J. Immunol..

[77] Domingo E. (2015). Introduction to virus origins and their role in biological evolution. Virus as Populations.

[78] Banerjee N., Mukhopadhyay S. (2016). Viral glycoproteins: Biological role and application in diagnosis. Virusdisease.

[79] Sadraeian M., Junior F.F.P., Miranda M., Galinskas J., Fernandes R.S., da Cruz E.F., Fu L., Zhang L., Diaz R.S., Cabral-Miranda G. (2022). Study of Viral Photoinactivation by UV-C Light and Photosensitizer Using a Pseudotyped Model. Pharmaceutics.

[80] Moore P.S., Chang Y. (2010). Why do viruses cause cancer? Highlights of the first century of human tumour virology. Nat. Rev. Cancer.

[81] Boshart M., Gissmann L., Ikenberg H., Kleinheinz A., Scheurlen W., zur Hausen H. (1984). A new type of papillomavirus DNA, its presence in genital cancer biopsies and in cell lines derived from cervical cancer. EMBO J..

[82] Durst M., Gissmann L., Ikenberg H., zur Hausen H. (1983). A papillomavirus DNA from a cervical carcinoma and its prevalence in cancer biopsy samples from different geographic regions. Proc. Natl. Acad. Sci. USA.

[83] Divya C.S., Pillai M.R. (2006). Antitumor Action of Curcumin in Human Papillomavirus Associated Cells Involves Downregulation of Viral Oncogenes, Prevention of NFΚB and AP-1 Translocation, and Modulation of Apoptosis. Mol. Carcinog..

[84] Vélez-Bohórquez A., Bohórquez-Lozano M., Echeverry-de-Polanco M. (2018). The virus in the Human oncogenesis. Infectio.

[85] Bouza E., Jiménez M.M., Alemany L., Arribas J., Bañares R., Barragán M.B., Eiros Bouza J.M., Felip E., Fernández-Capetillo O., Gracia D. (2021). Overview of virus and cancer relationships. Position paper. Rev. Esp. Quimioter..

[86] Passos A.M., Granato C.F.H. (2013). Cancer causing viruses and the role of laboratory medicine: Literature review and perspectives. J. Bras. Patol. Med. Lab..

[87] Epstein M.A., Achong B.G., Barr Y.M. (1964). Virus particles in cultured lymphoblasts from Burkitt’s lymphoma. Lancet.

[88] Chang Y., Cesarman E., Pessin M.S., Lee F., Culpepper J., Knowles D.M., Moore P.S. (1994). Identification of herpesvirus-like DNA sequences in AIDS-associated Kaposi’s sarcoma. Science.

[89] Dane D.S., Cameron C.H., Briggs M. (1970). Virus-like particles in serum of patients with Australia-antigen-associated hepatitis. Lancet.

[90] Soyama T., Sakuragi A., Oishi D., Kimura Y., Aoki H., Nomoto A., Yano S., Nishie H., Kataoka H., Aoyama M. (2021). Photodynamic therapy exploiting the anti-tumor activity of mannose-conjugated chlorin e6 reduced M2-like tumor-associated macrophages. Transl. Oncol..

[91] Kim H.J., Yoo H.S., Kim J.C., Park C.S., Choi M.S., Kim M., Choi H., Min J.S., Kim Y.S., Yoon S.W. (2009). Antiviral effect of *Curcuma longa* Linn extract against hepatitis B virus replication. J. Ethnopharmacol..

[92] Steinmann E., Gravemann U., Friesland M., Doerrbecker J., Müller T.H., Pietschmann T., Seltsam A. (2013). Two pathogen reduction technologies – methylene blue plus light and shortwave ultraviolet light—Effectively inactivate hepatitis C virus in blood products. Transfusion.

[93] Blumberg B.S., Alter H.J., Visnich S.A. (1965). “new” antigen in leukemia sera. JAMA.

[94] Kim K., Kim K.H., Kim H.Y., Cho H.K., Sakamoto N., Cheong J.H. (2010). Curcumin inhibits hepatits C virus replication via suppressing the Akt-SREBP-1 pathway. FEBS Lett..

[95] Zhang B., Zheng L., Huang Y., Mo Q., Wang X., Qian K. (2011). Detection of Nucleic Acid Lesions During Photochemical Inactivation of RNA Viruses by Treatment with Methylene Blue and Light Using Real-time PCR. Photochem. Photobiol..

[96] Hoofnagle J.H. (2002). Course and outcome of hepatitis C. Hepatology.

[97] Yoshida M., Miyoshi I., Hinuma Y. (1982). Isolation and characterization of retrovirus from cell lines of human adult T-cell leukemia and its implication in the disease. Proc. Natl. Acad. Sci. USA.

[98] Seiki M., Hattori S., Hirayama Y., Yoshida M. (1983). Human adult T-cell leukemia virus: Complete nucleotide sequence of the provirus genome integrated in leukemia cell DNA. Proc. Natl. Acad. Sci. USA.

[99] Poiesz B.J., Ruscetti F.W., Gazdar A.F., Bunn P.A., Minna J.D., Gallo R.C. (1980). Detection and isolation of type C retrovirus particles from fresh and cultured lymphocytes of a patient with cutaneous T-cell lymphoma. Proc. Natl. Acad. Sci. USA.

[100] Oka T., Matsuoka K.I., Utsunomiya A. (2020). Sensitive Photodynamic Detection of Adult T-cell Leukemia/Lymphoma and Specific Leukemic Cell Death Induced by Photodynamic Therapy: Current Status in Hematopoietic Malignancies. Cancers.

[101] Xu L., Zhang X., Cheng W., Wang Y., Yi K., Wang Z., Zhang Y., Shao L., Zhao T. (2019). Hypericin-photodynamic therapy inhibits the growth of adult T-cell leukemia cells through induction of apoptosis and suppression of viral transcription. Retrovirology.

[102] Akula S.M., Pramod N.P., Wang F.Z., Chandran B. (2002). Integrin alpha3beta1 (CD 49c/29) is a cellular receptor for Kaposi’s sarcoma-associated herpesvirus (KSHV/HHV-8) entry into the target cells. Cell.

[103] Feng H., Shuda M., Chang Y., Moore P.S. (2008). Clonal integration of a polyomavirus in human Merkel cell carcinoma. Science.

[104] Ikeda I., Yamashita Y., Ono T., Ogawa H. (1994). Selective phototoxic destruction of rat Merkel cells abolishes responses of slowly adapting type I mechanoreceptor units. J Physiol..

[105] Sixbey J.W., Nedrud J.G., Raab-Traub N., Hanes R.A., Pagano J.S. (1984). Epstein-Barr virus replication in oropharyngeal epithelial cells. N. Engl. J. Med..

[106] Robinson W.S., Clayton D.A., Greenman R.L. (1974). DNA of a human hepatitis B virus candidate. J. Virol..

[107] Kelly G.L., Rickinson A.B. (2007). Burkitt lymphoma: Revisiting the pathogenesis of a virus-associated malignancy. Am. Soc. Hematol. Educ. Program.

[108] Porta C., Paglino C., Mosca A. (2014). Targeting PI3K/Akt/mTOR signaling in cancer. Front. Oncol..

[109] Dowling R.J., Topisirovic I., Alain T., Bidinosti M., Fonseca B.D., Petroulakis E., Wang X., Larsson O., Selvaraj A., Liu Y. (2010). mTORC1-mediated cell proliferation, but not cell growth, controlled by the 4E-BPs. Science.

[110] Bossler F., Hoppe-Seyler K., Hoppe-Seyler F. (2019). PI3K/AKT/mTOR Signaling Regulates the Virus/Host Cell Crosstalk in HPV-Positive Cervical Cancer Cells. Int. J. Mol. Sci..

[111] Serrano M., Lin A.W., McCurrach M.E., Beach D., Lowe S.W. (1997). Oncogenic ras provokes premature cell senescence associated with accumulation of p53 and p16INK4a. Cell.

[112] Kim E.K., Choi E.J. (2015). Compromised MAPK signaling in human diseases: An update. Arch. Toxicol..

[113] Bourotto M., Chiou V.L., Lee J.M., Elise C., Kohn M. (2014). The MAPK pathway across different malignancies: A new perspective. Cancer.

[114] Lobry C., Oh P., Aifantis I. (2011). Oncogenic and tumor suppressor functions of Notch in cancer: It’s NOTCH what you think. J. Exp. Med..

[115] Nusse R., Clevers H. (2017). Wnt/β-catenin signaling, disease, and emerging therapeutic modalities. Cell.

[116] Wang B., Li X., Liu L., Wang M. (2020). β-Catenin: Oncogenic role and therapeutic target in cervical cancer. Biol. Res..

[117] Zhang Q., Lenardo M.J., Baltimore D. (2017). 30 Years of NF-κB: A blossoming of relevance to human pathobiology. Cell.

[118] Sun S.C., Cesarman E. (2011). NF-κB as a target for oncogenic viruses. Curr. Top. Microbiol. Immunol..

